# Inflammation and Related Signaling Pathways in Acute Myeloid Leukemia

**DOI:** 10.3390/cancers16233974

**Published:** 2024-11-27

**Authors:** Nour Sabiha Naji, Mrudula Sathish, Theodoros Karantanos

**Affiliations:** 1 Department of Oncology, Johns Hopkins School of Medicine, Baltimore, MD 21205, USA; nnaji1@jh.edu; 2 Department of Pediatrics, Albert Einstein College of Medicine, Bronx, NY 10461, USA; mrudula.sathish@einsteinmed.edu

**Keywords:** acute myeloid leukemia, inflammation, clonal hematopoiesis, immune tolerance

## Abstract

Acute myeloid leukemia (AML) is an aggressive hematologic malignancy, and inflammatory signaling is involved in its pathogenesis. Cytokines exert a robust effect on the progression of AML and affect survival outcomes. The dysregulation in the cytokine network may foster a pro-tumorigenic microenvironment, increasing leukemic cell proliferation, decreasing survival and driving drug resistance. The dominance of pro-inflammatory mediators such as IL-11β, TNF-α and IL-6 over anti-inflammatory mediators such as TGF-β and IL-10 has been implicated in tumor progression. Additionally, inflammatory cytokines have favored certain populations of hematopoietic stem and progenitor cells with mutated clonal hematopoiesis genes. This article summarizes current knowledge about inflammatory cytokines and signaling pathways in AML, their modes of action and the implications for immune tolerance and clonal hematopoiesis, with the aim of finding potential therapeutic interventions to improve clinical outcomes in AML patients.

## Simple Summary

Ongoing research has studied the implication of inflammation in acute myeloid leukemia (AML). This link can suggest potential therapeutic inflammatory targets which may improve prognosis in this disease. The aim of this review is to consolidate knowledge on the association of inflammation and hematologic malignancies, particularly AML, providing insight on the involvement of inflammation in immune surveillance and clonal hematopoiesis.

## 1. Introduction

Acute myeloid leukemia (AML) is an aggressive hematologic malignancy originating from stem and progenitor myeloid cells. It is pathologically marked by abnormal proliferation, suppressed differentiation, and the clonal expansion of myeloid cells within the bone marrow [1]. The onset of AML may result from genetic changes, including chromosomal alterations or individual gene mutations, leading to an expansion of neoplastic clonal myeloid stem cells [2]. Genetic mutations are found in over 97% of cases, even without the detection of any large chromosomal abnormalities such as well-described translocations. Molecular changes are also key contributors to the development of AML [3]. One of the best-described molecular alterations that act as key contributors to AML pathogenesis is the overexpression of tumor necrosis factor (TNF), which promotes leukemic cell survival and proliferation and is thus associated with inflammation in AML and poor prognosis [4]. Another vital molecular alteration that contributes to AML is the activation of the nuclear factor-kappa-B (NF-Κb) pathway, which is a regulator of inflammation and immune response, leading to the overproduction of inflammatory cytokines supporting AML proliferation [5]. Recently, dysregulation of myeloid-derived suppressor cells (MDSCs), a population of heterogeneous cells that promote tumor growth and simultaneously suppress immune response, are increased in AML and have been linked to inflammation and poor patient prognosis [6].

Notably, leukemogenic agents, such as certain pro-inflammatory cytokines and some immunomodulators, induce sustained inflammation and might lead to chronic conditions that promote cancer, including angiogenesis, tumor proliferation and tumor invasiveness [7]. Inflammation, a hallmark of cancer [8], has been associated not only with solid tumors but also with the development of myelodysplastic syndrome (MDS) to AML [9] and plays a fundamental role in altering the composition of the cancer microenvironment [10]. AML primarily affects older adults, and age-related inflammation might play a role in its development. In AML, the immune system responds to the inflammatory environment differently than in solid tumors, and this also holds true for the immune activation at a systemic level [11]. While inflammation is a factor in both solid tumors and AML, its systemic nature in AML requires different considerations for treatment and patient response [6].

Meanwhile, the impact of inflammation on the bone marrow immune microenvironment and clinical outcomes in AML remains unclear. Efforts to target immune cell functions in AML have had limited success, underscoring the need for a deeper understanding of the AML immune microenvironment and its interaction with clonal hematopoiesis, which is crucial for understanding the disease progression and the role of inflammation [12,13,14]. The purpose of this review article is to highlight the role of inflammatory mediators and inflammatory signaling in AML and describe its impact on immune tolerance, disease progression and resistance to treatments.

## 2. Impact of Inflammation on Clonal Hematopoiesis

Clonal hematopoiesis (CH) refers to the clonal expansion of hematopoietic stem and progenitor cells (HSPCs) [15]. Its prevalence rises significantly with age, and it usually results from mutations in specific genes or from gains and/or losses of larger chromosomal regions [16]. It is considered a premalignant condition, as these clones can act as ancestor clones during the progression to hematologic malignancies, making CH a strong indicator of the risk of developing blood cancers [17,18]. Mutations detected in DNMT3A, TET2 and ASXL1 comprise approximately 80% of all detected mutations, with DNMT3A making up almost 60% of the total mutational burden [19,20,21]. Other commonly detected mutations include JAK2, TP53, PPM1D, SF3B1 and SRSF2 [20].

Clonal hematopoiesis can be linked to cardiovascular diseases. Interestingly, anti-inflammatory therapies are efficient at treating these diseases, notably the therapies targeting inflammasomes, downstream targets like interleukin-1beta (IL-1β) and interleukin-6 (IL-6), or mutations associated with clonal hematopoiesis [22]. Lately, CH has also been linked to various blood disorders, which has led to the introduction of many new definitions and acronyms that include CH [23]. Clonal hematopoiesis of indeterminate potential (CHIP) is a term used to describe the absence of malignancy or cytopenia or other defined clonal disorders, such as monoclonal gammopathy of undetermined significance, and provokes the requirement that the variant allele frequency (VAF) for a clone be ≥2% [24,25].

The association between inflammation and CH is complex. In fact, in humans, CH is linked to elevated levels of interleukin-6 (IL-6), interleukin-8 (IL-8) and TNF, with specific gene–cytokine associations, particularly *TET2* mutations with IL-6 and *DNMT3A* with TNF-*α* [26]. Importantly, preclinical studies in mice have revealed that HSPC with *Tet2* and *Dnmt3a* loss-of-function mutations exhibit higher proliferation when exposed to certain inflammatory signals [27,28]. In a mouse model of mutant *DNMT3A* HSPCs, TNF receptors (TNFR1 and TNFR2) were necessary to sustain the mutant cells’ proliferative advantage and lineage specificity. Serial transplantation experiments, followed by transcriptomic analyses, revealed that decreased TNFR1 led to lower clonal fitness while preserving balanced lineage output. In contrast, TNFR2 played a key role in promoting lymphoid maturation and dominance of mature B cells [29]. Collectively, these findings indicate that inflammatory cytokines play a role in favoring certain populations of HSPCs with mutated CH genes, and that, for each identified mutation, there are distinct cytokine-specific pathways at play as well as consequent changes in gene expression that enhance the inflammatory response.

## 3. T Cell Immunity and Immune Tolerance in Acute Myeloid Leukemia

Immune surveillance mechanisms include both the adaptive and innate immune systems which are naturally present to prevent AML development. However, leukemic cells eventually escape immune surveillance and result in tumor progression [30]. Several mechanisms are involved in AML’s immune evasion, contributing to the immune tolerance of leukemic cells include:

### 3.1. Altered Antigen Presentation

Leukemia blasts demonstrate significant ability to affect immune activity when exposed to selective immune pressure following allogeneic hematopoietic stem cell transplantation [31], as shown by the loss of mismatched HLAs in haploidentical transplants [32,33,34] and the epigenetic downregulation of HLA class II molecules in various donor transplant cases [35,36]. In AML mouse models, leukemia antigen presentation by immature antigen-presenting cells or splenic CD8a+ dendritic cells (DCs) have respectively induced deletional T cell tolerance and CD8+ T cell tolerance [37,38]. Additionally, inferior survival outcomes and measurable residual disease after AML treatment were associated with loss of plasmacytoid DC differentiation [39].

### 3.2. T and NK Cells

The presence of T cells at the tumor site is essential for immune recognition and elimination of AML cells. Compared with healthy controls (HCs), patients with AML have a similar quantity of T cells in the bone marrow but show increased levels of total and CD8+ T cells in peripheral blood [40,41]. Elevated percentages of lymphocytes, and particularly T lymphocytes, in bone marrow were associated with better response and survival [42], and lymphocyte recovery after chemotherapy was correlated with a lower risk of relapse [43]. This is particularly helpful in predicting responses to therapy; higher percentages of CD3+ and CD8+ T cells in the bone marrow, for example, were shown to predict response to the checkpoint inhibitor nivolumab in combination with a hypomethylating agent [44].

In AML, T cell dysfunction is at least partly due to an imbalance in the cytokine network and impaired immunological synapses between activated T cells and AML blasts [45]. Infiltration of cytotoxic lymphocytes was found to be associated with TP53 and myelodysplastic-related changes in AML [46]. A recent study has indicated that Th1 cells may have limited functionality, as Th1-derived IFN-γ levels are significantly lower in peripheral blood mononuclear cells (PBMCs) from AML patients following stimulation with phorbol myristate acetate and ionomycin [47]. The bone marrow plasma of AML patients consistently exhibits significantly lower levels of IFN-γ when compared with controls [48]. T helper type 17 (Th17) cells, another subset of CD4+ Th cells, have been reported to be significantly increased in AML patients, alongside a notable decrease in the CD4+ Th1 cell population in both bone marrow and peripheral blood. The combination of elevated Th17 and reduced Th1 is associated with poor prognosis in AML. In fact, the differentiation of naïve T cells toward Th17 necessitates a combination of at least two of the following cytokines: IL-1β, IL-6 or Il-23 or all of the latter cytokines together [49]. Additionally, TGF-β1, a key cytokine for Th17 differentiation in mice models [50], resulted in a decrease in the Th17 population [49] and did not correlate with the frequencies of Th17 cells in the peripheral blood of untreated AML patients; this further supports the fact that TGF-β may hold protective effects in AML [51]. Interleukin-17A (IL-17A), a characteristic cytokine of Th17 cells, induces proliferation of primary AML cells due to high expression of IL-17A receptor (IL-17R) on the cell surface. Only AML patients with complete remission after chemotherapy had lower proportions of Th17 cells following treatment [52].

### 3.3. Immunosuppressive Microenvironment

Murine and human studies have shown that Tregs contribute to defective immune responses in AML [53,54]. The latter is mainly due to the high proportion of Tregs in peripheral blood and bone marrow of AML patients [55], which show stronger immunosuppressive effects on effector T cells than normal cells and are largely unaffected by chemotherapy [41]. Altogether, this results in a correlation between Treg levels and poor outcomes in AML patients [56]. The immune microenvironment further contributes to what cancer types respond to immune targeting. For example, a study by Vadakekolathu et al. showed that interferon-gamma (IFN-γ)-related mRNA profiles predicted chemotherapy resistance and the response of primary refractory/relapsed AML to flotezumab immunotherapy [57]. In addition, oncogenic drivers also define AML’s immune response and further have an effect in immune escape. In particular, Nras^G12D^ has been shown to induce an anti-leukemic response in AML through increased MHC class II expression that can be surpassed with increased Myc expression [58,59].

### 3.4. Myeloid-Derived Suppressor Cells (MDSCs)

MDSCs are a heterogeneous group of immunosuppressive myeloid cells which originate from a common myeloid progenitor and appear to be increased in AML patients [60,61]. Meanwhile, the specific processes that promote MDSC expansion and how they function in the immunosuppressive microenvironment of myeloid malignancies is not well understood [62]. Preclinical studies in AML cell lines and murine models have shown that MDSCs suppress T cell responses and T cell proliferation and that the MUC-1-mediated expression of c-myc drives MDSC expansion [63]. Particularly, AML cells release extracellular vesicles containing the oncoprotein MUC-1, which increases c-myc expression in extracellular vesicles and leads to MDSC proliferation [63,64]. A positive correlation between the number of Tregs and MDSCs has been reported in high-risk MDS, suggesting the potential role of both Tregs and MDSCs in disease progression [65].

## 4. Role of Pro-Inflammatory Mediators in AML

Among the key pro-inflammatory cytokines impacting the function of hematopoietic cells are TNF-α, interleukin-1 (IL-1) and interleukin-6 (IL-6) [66]. Dysregulation of cytokine expression, a hallmark of chronic inflammation, is a feature frequently described during hematologic malignancy development and progression [52]. The role of cytokines has been analyzed by Carey et al., who identified the cytokines and growth factor pathways critical for AML cell survival [67]. Using an ex vivo cell viability screen, they quantified the growth of 60 primary AML samples in the presence of 94 different cytokines. AML cell growth significantly increased in the presence of IL-1α, granulocyte-macrophage colony-stimulating factor (GM-CSF), interleukin-3 (IL-3) and TNF-α. However, the most prominent effect was seen with exogenously added IL-1β, with up to a 15-fold increase in cell growth and survival in almost 70% of the primary AML samples. The following inflammatory cytokines have the greatest effect on AML cell survival [67].

### 4.1. Interleukin-1 (IL-1)

IL-1 has two different forms which exert their pro-inflammatory activity by binding to IL-1R1: IL-1α and IL-1β [68]. IL-1α is constitutively expressed in healthy individuals and induces multiple signal transduction pathways in sterile inflammation [69,70]. However, IL-1β is an essential cytokine of both the innate and adaptive immune systems [71]. The main source of IL-1β is cells of the myeloid branch, particularly activated macrophages, monocytes and dendritic cells [72]; however, it is also expressed in nonhematopoietic cells such as endothelial cells and fibroblasts [73]. Increased production of IL-1β has been described in hematological malignancies and has been recognized as a biomarker for unfavorable prognosis in AML patients. Thus, it is important to understand its regulation and signal transduction network [74,75]. To particularly highlight the role of IL-1β/IL-1R1 signaling in AML, Carey et al. showed that siRNA knockdown of IL-1R in primary AML cells as well as in a murine AML transplantation model induced survival and AML cell growth, further worsening disease progression independently of mutational status. Upon IL-1β exposure, hematopoietic stem cells suffer significant loss of self-renewal ability and may even have a negative effect on growth; however, IL-1β plays a crucial role in survival for leukemic cells [67]. Therefore, IL-1β and components of the IL-1β signaling pathway may be promising targets for future therapeutic agents.

### 4.2. Interleukin-6 (IL-6)

Understanding the role of IL-6 in human diseases has prompted the development of innovative treatments that target and block the biological functions of IL-6 [76]. Clinically, IL-6 and IL-6 receptor antibodies have proven to have great efficacy in conditions that are driven by IL-6 such as rheumatoid arthritis [77], systemic juvenile arthritis [78] and Castleman’s disease [79]. Besides its role as an acute phase protein in inflammation, IL-6 is a key pleotropic cytokine that regulates hematopoiesis and the formation of leukemic blasts [80]. Although IL-6 levels have been noted to be elevated in AML patients [81,82], it appears to have opposed effects, and thus, its benefit as a predictive marker or therapeutic target is still not validated. IL-6 promoted growth of blast cells and increased GM-CSF-dependent proliferation in some patients [83,84], while in others it had no similar effect [85]. IL-6 should be thus further studied to identify its utility in AML and its impact on the responsiveness of leukemic cells.

### 4.3. Interleukin-8 (IL-8)

Aberrant overexpression of IL-8 has been reported in several myeloid malignancies, such as MDS, AML and myeloproliferative neoplasms (MPNs) [86]. IL-8 (CXCL8) is a CXC chemokine usually secreted by abnormal hematopoietic stem and progenitor cells. This cytokine binds to CXCR1/CXCR2 receptors and promotes downstream oncogenic signaling pathways, recruiting myeloid derived suppressor cells to the tumor microenvironment [87]. Thus, high CXCR2 expression in HSPCs has been correlated with adverse prognosis in AML and MDS. In line with the latter, CCRL2, an atypical chemokine receptor which dimerizes with CXCR2, is upregulated in MDS and secondary AML, and CCRL2 plays a role in cell growth and clonogenicity, partially through JAK2/STAT signaling [88]. Additionally, CCRL2 targeting seems to have a therapeutic potential as CCRL2 modulates epigenetic regulatory pathways, particularly DNMT levels, and affects the sensitivity of MDS/secondary AML cells to azacitidine [89].

### 4.4. Tumor Necrosis Factor-Alpha (TNF-α)

TNF-α presents in two distinct bioactive forms, membrane-bound TNF-α (tmTNF-α) which is enzymatically converted by metalloproteases into its secretory form (sTNF-α) [90]. TmTNF-α has been implicated in the proliferation of leukemic stem cells (LSCs) and adverse clinical outcomes in acute leukemia [91]. Particularly in AML, TNF-α is implicated in drug resistance and enhanced survival of leukemic clones [92]. Meanwhile, exposure of normal hematopoietic stem cells to TNF-α may lead to growth inhibition [93,94,95,96]. The contrasting roles of cytokines has been a typical feature of hematological malignancies, including AML. As the elevated levels of tmTNF-α seem to be restricted to LSCs, it may be effective to target tmTNF-α to eradicate LSCs. Recent findings have demonstrated that most AML cells express both TNF-α and IL-1β, so the combined inhibition of TNF-α and IL-1β has been shown to synergize with NF-κB, effectively eliminating the LSC pool ex vivo and exhibiting significant anti-leukemic activity in in vivo mouse models [97].

### 4.5. Interferon Gamma (IFN-γ)

IFN-γ is secreted by activated T cells and plays an important role as a mediator between innate and adaptive immune systems. IFN-γ is a known pro-inflammatory cytokine that promotes anti-leukemic activity [98]. Low serum IFN-γ levels correlated with a higher percentage of LSCs and greater incidence of AML relapse. Specifically, high doses (5–10 μg/day) of IFN-γ exhibited an anti-AML effect, whereas low doses (0.01–0.05 μg/day) accelerated AML development and promoted the self-renewal of LSCs in patient-derived AML-LSCs as well as in a mouse model enriched for LSCs driven by MLL-AF9 [99]. IFN-γ signaling has contributed to a unique microenvironment in monocytic and del7/7q subtypes and has been significantly associated with negative prognosis [100]. Meanwhile, IFN-γ signaling score is strongly linked with venetoclax resistance in primary AML patients and, upon IFN-γ treatment, primary AML patient cells increased venetoclax resistance [100]. Thus, inhibiting IFN-γ can be a potential therapeutic mechanism to overcome venetoclax resistance and restore immune surveillance in AML. Notably, targeting the IFN-γ receptor IFNGR1 through lentiviral shRNAs or neutralizing antibodies facilitated AML differentiation and delayed leukemogenesis in vitro and in murine models [99]. Additionally, anti-leukemic effects have been reported for IFN-γ even in the presence of IL-1β and growth promoting factors such as GM-CSF, G-CSF or human stem cell factor (SCF) as the growth inhibitory effect of IFN-γ is dependent on the cytokine network rather than a reduction of IL-1β or growth factor levels [101]. Malignant blasts from AML patients have shown to secrete type I interferon, which exerts a cytostatic and cytotoxic effect, contributing to treatment efficacy in those patients and affecting prognosis and overall survival in AML patients [102]. Additionally, inflammation promotes *TP53*-associated clonal dominance, and *TP53* heterozygous hematopoietic stem cell/progenitor cells from pre-*TP53*-secondary AML showed the increased expression of oxidative phosphorylation, DNA repair and interferon response genes without further modifications in levels of IFN receptor expression or concurrent IFN treatment [103].

## 5. Inflammatory Pathways in Acute Myeloid Leukemia

**Figure 1:**
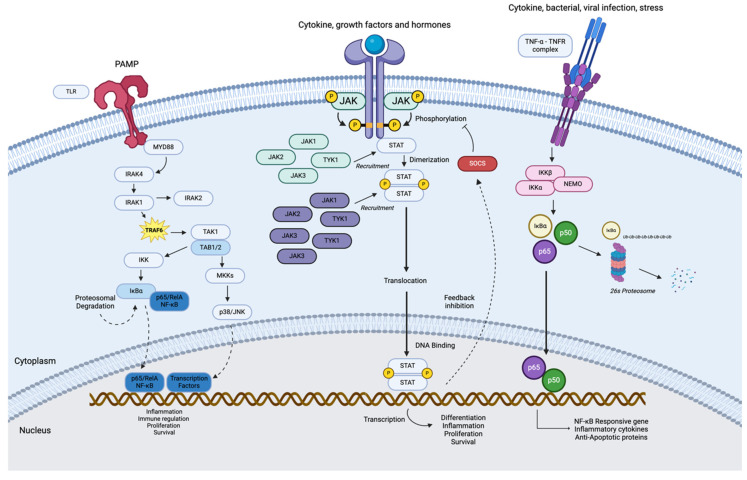
A summary of the inflammatory pathways implicated in AML pathogenesis.

Several inflammatory pathways have been closely linked to AML pathogenesis and have had a crucial impact on prognosis and survival outcomes [6]. The dysregulation in these pathways has been implicated in hematopoietic defects in the bone marrow microenvironment. Among these pathways are those discussed below, and these pathways are summarized in Figure 1.

### 5.1. Toll-Like Receptor (TLR) Signaling Pathway

The innate immune system plays a crucial role in defending our bodies from various pathogens and cancer by identifying pathogen-associated molecular patterns (PAMPs) and damage-associated molecular patterns (DAMPs). Toll-like receptors (TLRs) are a family of pattern recognition receptors, and are found in many immune cells like dendritic cells, neutrophils and even macrophages [104].

TLR signaling pathway works by initiating cascades that produce pro-inflammatory cytokines and type 1 interferons, post binding to a ligand [105]. TLR signaling pathway is key to the pathogenesis of AML because it promotes survival, proliferation, and differentiation of leukemic cells. It activates various downstream pathways, including NF-κB and MAPK, the most prominent targets in AML [106]. The latter leads to the production of inflammatory cytokines, supporting the growth and survival of leukemic cells, and it further interacts with other pathways that enhance leukemic cell proliferation, such as FMS-like tyrosine kinase 3 pathway which is activated by the internal tandem duplications (FLT3-ITDs) [107]. During TLR activation in AML, adaptor proteins, like MyD88 and TRIF4, are recruited due to the recognition of PAMPs and DAMPs involved [108]. Meanwhile, downstream kinase targets—IRAK1, IRAK4 and TRAF6—are triggered as a response to the recruitment of adaptor proteins, activating NF-κB and MAPK pathways [106].

However, TLR agonists are used as promising therapeutic approaches in AML by improving immune response, strategically activating these pathways to direct the immune system’s confrontation with the leukemic cells. An increase in anti-leukemic effects were observed with TLR7/8 agonists promoting T cell activation and proliferation [109]. In addition to TLR 7/8, TLR 9 agonists have also been proven to have the potential to induce cell death in AML [107]. The dual role of TLR signaling in AML is conflicting in a way that TLR signaling pathway fosters the growth and survival of leukemic cells, but with the right agonist treatment, a shift towards anti-leukemic effects by enhancement of immune responses can be observed.

Finally, studies highlight that TLR-targeted therapies show promise in enhancing AML patient outcomes. Progressing research is now focused on developing new TLR agonists and creating combination therapies that affect several signaling pathways [109]. Moreover, exploring TLRs within the tumor microenvironment and their interactions with immune cells could unlock new therapeutic possibilities.

### 5.2. NF-κB Pathway

Nuclear factor-κB (NF-κB) is mediated by two main signaling pathways, the canonical and non-canonical pathways, which are different in terms of signaling mechanisms and biological functions [110]. The action of RelA/p50 subunits in the cytoplasm is the key mediator for the more common, the canonical or classical NF-κB pathway. Ubiquitinated proteosome activity degrades the physiological inhibitor of NF-κB, IκBα, which is also present in the cytoplasm, after which the p50/RelA subunits, forming a complex, translocate into the nucleus and begin transcription of the target genes. The IκB–kinase (IKK) complex phosphorylates IκBα, causing its breakdown and activating this pathway. The non-canonical or alternative pathway, on the other hand, depends on the activation of the RelB subunit linked to either p50 or p52. This pathway is triggered by ligands, like CD40L, lymphotoxin β (LTβ), receptor activator nuclear factor-κB ligand, or B cell-activating factor, that belong to the TNF family through NF-κB inducing kinase (NIK), which activates IKKα and causes the breakdown of p100, an IκB-like molecule and a precursor of the p52 subunit [111,112]. Following the breakdown of p100, RelB/p50 and RelB/p52 enter the nucleus and begin the target genes’ transcription [113].

TNF-R-associated factors (TRAFs) play crucial roles in both the canonical and non-canonical NF-κB signaling pathways and act upstream of IKK or NIK activation, respectively. Notably, TRAF2 and TRAF3 exhibit distinct signaling functions. The recruitment of TRAF2 and TRAF6 is essential for activating the canonical pathway, while TRAF3 does not contribute directly to this pathway, instead, it negatively regulates TRAF2 and TRAF6 and plays a major role in the noncanonical pathway [114].

Blast numbers, apoptosis suppression, and disease progression were all found to be linked to RelA activation in 57 samples of MDS patients [107]. Bone marrow cell death was caused by suppression of the classical route by siRNA, anti-RelA, or Bortezomib, which prevents the proteasomal breakdown of IκBα and p100 [111]. Likewise, another investigation on AML patients confirmed that RelA/p50 and p50/p50 complexes, which actively function in AML cells, are examples of the canonical pathway [115]. In AML, FLT3-ITD activates the non-canonical pathway mediated by RelB/p52. RelB/p52 can inhibit the tumor suppressor death-associated protein kinase 1 (DAPK1) and promote tumor development [116].

By targeting the TNF-α/NF-κB pathway, prominently using NF-κB inhibitors and TNF-α antagonists, or even inhibiting their downstream targets, like IKK inhibitors, Bcl2 inhibitors or MDM2 inhibitors can serve as a potential therapeutic option in AML. They not only disrupt signals of pro-survival but also induce apoptosis in leukemic cells sensitizing AML to chemotherapy and consecutively improving prognosis [117]. The development of new NF-κB inhibitors and combination treatments that target various signaling pathways are emerging research areas. Furthermore, research is underway that examines TNF-α’s function in the tumor microenvironment and how it interacts with other immune cells, which may result in novel treatment approaches.

### 5.3. JAK/STAT Pathway

The Janus-associated kinase-signal transducers and activators of transcription (JAK/STAT) signaling pathway is a common intracellular signal transduction pathway implicated in vital biological processes, including cell proliferation, differentiation, apoptosis and immune regulation [118]. The JAK/STAT pathway is particularly important in the study of cancers, because it transmits information from the extracellular signals to the nucleus, impacting a range of cellular processes like survival, heterogeneity, immune response and even cell growth [119]. Current research on this pathway focuses on the inflammatory and neoplastic disease implications [120].

Almost two-thirds of AML patients show hyperactivate signal transduction pathways, including the JAK/STAT pathway [121]. Although gain-of-function JAK2V1617F is commonly seen in MPNs [122,123], mutated JAK2 is noted in <3% of de novo AML patients [124]. Meanwhile 44–100% of bone marrow samples from AML patients have been found to have elevated levels of phosphorylated JAK2, STAT3 and STAT5 in vitro [125,126]. Recent studies have highlighted various previously unknown activators of JAK2/STAT signaling in MDS and AML that do not also activate mutations in this pathway [88,89].

The collaborative activation of JAKs and STATs in AML promotes the growth and survival of leukemic cells. If the pathway is deregulated, it may not only activate unhindered cell proliferation in AML stem and progenitor cells, but also induce resistance to apoptosis [127], simultaneously interacting with other pathways, like PI3K/AKT and RAS/RAF/MEK/ERK, and creating a complex network that promotes leukemogenesis [128,129]. There is also reported crosstalk of the JAK/STAT pathway with other tyrosine kinase pathways. For example, treatment with combined JAK2 and FLT3 inhibitors has been found to decrease leukemic cell proliferation in vitro [130] and to promote leukemic regression in vivo [131].

Although the role of JAK inhibitors has not been very effective in AML, JAK inhibitors, like ruxolitinib, have shown efficacy in MPN subtypes such as polycythemia vera and myelofibrosis [121,132,133,134]. Rampal et al. used a Tp53 KO/Jak2V617F leukemic mouse model to evaluate the efficacy of ruxolitinib in MPNs and its use in combinatorial therapies in vitro and in vivo [135]. Ruxolitinib or decitabine monotherapy has been found to result in a concentration-dependent inhibition of colony formation, while the combination of decitabine and ruxolitinib showed improved efficacy in vitro [121]. However, the clinical efficacy of JAK inhibitor monotherapy in AML has been very limited overall, suggesting that this pathway is likely not critical for the survival of leukemia blasts but potentially mediates disease progression and resistance to other therapies.

## 6. Role of Anti-Inflammatory Mediators in AML

**Figure 2:**
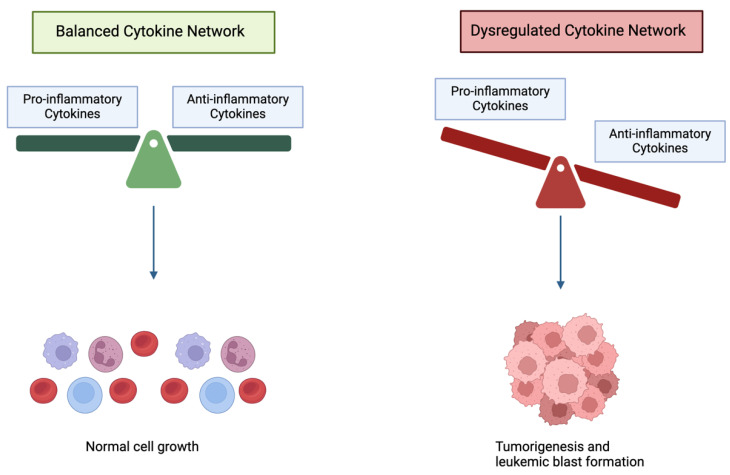
The effect of cytokine network dysregulation on tumorigenesis and leukemic blast formation.

Although the pro-inflammatory microenvironment is the hallmark of AML, anti-inflammatory cytokines, such as transforming growth factor beta (TGF-β) and interleukin-10 (IL-10), greatly contribute to the cytokine dysregulation implicated in AML progression and worsening survival (Figure 2) [136]. Among the most common anti-inflammatory mediators in AML are those summarized below:

### 6.1. Interleukin-1 Receptor Antagonist (IL-1Ra)

Unlike most other members of the IL-1 family, IL-1Ra functions as a negative regulator of inflammation. IL-1Ra binds to IL-1R1 and competitively blocks IL-α and IL-1β [137]. Anakinra, a recombinant IL-1Ra, is commonly used for the treatment of inflammatory diseases [138]. Upon treatment with IL-1Ra in AML, there has been a reported reduction in spontaneous clonogenicity of these cells in addition to the clonogenicity of the cells subsequent to exposure to the antagonist [139]. Additional studies have shown that elevated levels of the IL-1 receptor antagonist were associated with positive outcomes; AML patients who had low IL-1β levels combined with high IL-1RA were remarkably protected against leukemic relapse [140]. Further research should be conducted to study the impact of IL-1β targeting on relapse and survival in AML.

### 6.2. Transforming Growth Factor-Beta (TGF-β)

TGF-β is a cytokine that is integral to the regulation of various cellular and physiological processes, including cell proliferation, migration and survival. Based on its immunosuppressive functions, TGF-β plays a key role in regulating growth inhibition and differentiation of hematopoietic progenitor cells [141,142]. Several studies have shown that TGF-β possesses a dual role: acting as a tumor suppressor in premalignant cells and as a tumor promoter in carcinoma cells [143]. During the early stages of tumorigenesis, it plays the role of a tumor suppressor by inhibiting proliferation and promoting apoptosis through cyclin-dependent kinase (CDK) inhibitors and downregulated MYC expression [144,145,146]. The suppressive role of TGF-β on leukemic blasts has been described in a large age-independent population of AML patients with significantly lower levels of TGF-β [147]. TGF-β can limit the self-renewal capacity of leukemic blast progenitors and inhibit the growth of leukemic cells [148,149]. Enhanced leukemogenesis is associated with lower levels of TGF-β and dysregulation of the TGF-β signaling pathway [150]. It is suggested that TGF-β is capable of downregulating the expression of the surface receptors of some cytokines, such as IL-1, IL-3, IL-6, GM-CSF and SCF, or of interfere with the signaling cascades induced by those cytokines and growth factors [151,152,153,154]. Consequently, TGF-β’s downregulation or dysregulated signaling may lead to an imbalance in the cytokine network affecting cell proliferation, migration and survival.

### 6.3. Interleukin-10 (IL-10)

IL-10, an anti-inflammatory cytokine produced by activated immune cells, has been detected in leukemic cells in AML patients and has been associated with the escape of leukemia cells from immune surveillance [155,156]. It has been shown that patients with increased IL-10 plasma levels have higher survival rates and better chemotherapy response rate [157]. Increased levels of IL-10 correlate with increased TNF-α and IL-6 levels in AML patients [157,158]. In contrast with TNF-α and IL-6, IL-10 has been proposed to downregulate pro-leukemic cytokines, such as IL-1α, IL-1β, IL-6, GM-CSF and TNF-α, and thus inhibit AML cell growth [159,160,161]. However, like other cytokines, it may have opposing effects depending on its cellular context. A study conducted by Diaz de la Guardia et al. showed that there was a positive correlation between elevated IL-10 levels secreted by bone marrow-derived mesenchymal stem/stromal cells (BM-MSCs) and decreased survival of AML patients. This study also reports that levels of TNF-α, IFN-γ, IL-1β, IL-8 and IL-6 secreted by BM-MSCs were not different between AML patients and healthy controls [162]. Due to the release of high IL-10 levels, AML-derived BM-MSCs have been suggested to have a more potent immunosuppressive ability than BM-MSCs from healthy donors [163]. The latter may be partially due to IL-10-induced release of IL-1Ra [164]. Additionally, the suppression of G-CSF and GM-CSF by IL-10 leads to inhibition of leukemic blast cells’ autocrine growth [159].

### 6.4. Interleukin-35 (IL-35)

IL-35 belongs to the family of IL-12 cytokines and has been shown to be aberrantly expressed in AML [165]. IL-35 is mainly expressed by regulatory T cells (Tregs) rather than antigen-presenting cells (APCs), and it is capable of suppressing CD4+ and CD8+ T cell proliferation and to reduce the cytokine expression on T cells [166]. IL-35 expression was found to be significantly increased in the bone marrows as well as plasma of AML patients, which were positively correlated to Treg percentage [54,167,168]. Studies have shown that IL-35 can directly inhibit the growth of effector T cells and diminish their cytotoxic abilities [165]. Additionally, IL-35 increased the proportion of CD4+ Tregs, which are highly effective at suppressing effector T cells [169]. These findings suggest that IL-35 plays a role in the immune escape of AML blasts by actively suppressing T cell functions while enhancing Treg activity. The significant involvement of IL-35 in AML pathogenesis has been further supported by evidence that pre-treatment with IL-35 leads to decreased apoptosis and increased proliferation of AML cells, indicating that this cytokine also fosters the growth of AML blasts [168]. Therefore, unlike the anti-inflammatory cytokines IL-10 and TGF-β, IL-35 does not inhibit AML cell growth.

## 7. Therapeutic Approaches Targeting Inflammation and Inflammatory Pathways in AML

As AML is an aggressive hematological malignancy, with inflammation and inflammatory pathways playing an important role in its pathogenesis, there is a need for better understanding of possible therapeutic interventions. There are different ways to counteract this aspect, most of which can be grouped into anti-inflammatory drugs, immunotherapies or targeted therapies.

### 7.1. Anti-Inflammatory Drugs

The addition of JAK inhibitors, NF-κB inhibitors, and NLRP3 inflammasome inhibitors in induction regimens are currently considered. JAK inhibitors, like ruxolitinib, that inhibit JAK1 and JAK2, not only reduce the production of pro-inflammatory cytokines but also modulate the inflammatory microenvironment in AML [170]. Bortezomib, a proteasome inhibitor, is a key inhibitor that indirectly inhibits NF-κB activation by preventing the degradation of its inhibitor, IκB [171]. Parthenolide is a natural compound which directly inhibits NF-κB activity [172]. These agents are being investigated for their preclinical efficacy and potential to reduce inflammation and induce apoptosis in AML cells [172]. Recently, NLRP3 inflammasome, a multiprotein complex involved in the activation of inflammatory responses, has been studied as a therapeutic agent in AML. Inhibitors targeting the NLRP3 inflammasome, such as MCC950, have shown potential in modulating inflammation in AML. MCC950 inhibits the activation of the NLRP3 inflammasome, reducing the production of pro-inflammatory cytokines and promoting apoptosis in leukemic cells [6]. More recently, STING activation has become the hallmark of AML by triggering p53-independent apoptosis, and STING agonists have shown a synergistic effect with BH3-mimetics in TP53-mutant blood cancers [173].

### 7.2. Immunotherapies

Checkpoint inhibitors, such as pembrolizumab [174] and nivolumab [175], target immune checkpoints, such as PD-1 and CTLA-4, are often upregulated in cancer cells and contribute to immune evasion. By blocking these checkpoints, these inhibitors enhance the activity of T cells and promote an anti-tumor immune response. The latter inhibitors have been effective in the treatment of many solid malignancies; however, there has been no clinical effect indicating its potential towards treating AML [176,177,178,179].

Alternatively, chimeric antigen receptor (CAR)–T cell therapy involves the genetic modification of T cells to express CARs that target specific antigens on leukemic cells. CAR–T cell therapy has shown potential in targeting inflammatory pathways and modulating the immune microenvironment. Immunotherapies have shown promise in terms of the treatment of solid tumors [180]. Similarly, there has been evidence that proves that CAR–T cell therapy has been a key player in the treatment of lymphoid malignancies [181]; however, there is no documented activity in AML so far. Meanwhile, CAR–T cells can be engineered to target antigens associated with inflammation, such as CD123 and CD33, which are expressed in AML cells [182]. Ongoing research on the efficacy of CAR–T cell therapy in AML is being conducted, and CAR–T cell therapy targeting NKGD2 ligands and CD123 are undergoing clinical trials [183,184,185].

### 7.3. Targeted Therapies

Novel therapies and research are emerging to treat AML and can be grouped mostly into small molecule inhibitors, monoclonal antibodies, and epigenetic modulators. Small molecule inhibitors targeting specific inflammatory pathways are being actively investigated in AML. For example, inhibitors of the bromodomain and extra-terminal (BET) proteins, such as JQ1, have shown potential in terms of modulating inflammation and reducing leukemic cell proliferation. BET inhibitors disrupt the interaction between BET proteins and acetylated histones, leading to the downregulation of pro-inflammatory genes [186]. Consecutively, monoclonal antibodies targeting inflammatory cytokines and their receptors are being explored as potential therapies for AML. For instance, antibodies targeting IL-6 and its receptor (IL-6R), such as tocilizumab, have shown promise in modulating inflammation in AML [187]. IL-6 is a pro-inflammatory cytokine that plays a key role in the pathogenesis of AML. By blocking IL-6 signaling, monoclonal antibodies can reduce inflammation and inhibit leukemic cell proliferation [187]. Epigenetic regulators, such as DNA methyltransferase inhibitors, show significant efficacy in MDS and AML [188] and their activity is partially mediated by modulating inflammatory signaling [189]. Clinical trials investigating the use of small molecule inhibitors, monoclonal antibodies, and epigenetic modulators, in combination with other therapies for AML, are ongoing, with promising preliminary results for patients with prior poor prognoses [190].

## 8. Conclusions

Inflammation has been reported as a major factor implicated in the biology of clonal hematopoiesis and contributing to AML pathogenesis. The unbalanced expression of cytokines and dysregulations in signaling pathways have a crucial impact in disrupting the microenvironment in AML and leading to disease progression. The interactions of pro- and anti-inflammatory cytokines regulate the cellular activation profile. While the major pro-inflammatory cytokines, like IL-1β, TNF-α and IL-6, promote tumorigenesis, certain anti-inflammatory cytokines, such as TGF-β and IL-10, counteract leukemogenesis and high levels of those are correlated with better prognosis. Unraveling the specific roles of cytokines and growth factors as drivers in AML requires further investigations.

Several major signaling pathways are involved in AML pathogenesis, and thus their inhibition improves survival. The advent of checkpoint inhibitors, combinatorial approaches with targeted therapies, immunotherapies and other therapeutic options targeting these key inflammatory pathways offers promising research avenues.

In conclusion, a better understanding of the molecular mechanisms driving inflammation in AML will allow for the introduction and evaluation of novel therapeutic options in AML and thus improve the prognosis and survival outcomes in this disease.
